# Sudden Loss of Vision: An Insight into Rivaroxaban

**DOI:** 10.7759/cureus.4542

**Published:** 2019-04-25

**Authors:** Akriti G Jain, Mamoon Ur Rashid, Manmeet Grewal, Ishtiaq Hussain

**Affiliations:** 1 Internal Medicine, Florida Hospital, Orlando, USA; 2 Internal Medicine, Dayanand Medical College, Ludhiana, IND; 3 Gastroenterology, Cleveland Clinic Florida, Weston, USA

**Keywords:** novel anticoagulants, direct oral anticoagulants, intra-ocular pressure, visual loss, rivaroxaban

## Abstract

A retrobulbar hemorrhage is a rapidly worsening emergent condition characteristically associated with significant orbital or facial trauma. Spontaneous hemorrhage into the retrobulbar compartment is rare. If not managed in a timely manner, it can lead to permanent vision loss. Rivaroxaban is a directly acting oral anticoagulant (DOAC) that inhibits factor Xa and is widely used for stroke prevention in patients with non-valvular atrial fibrillation (AFib).

## Introduction

A spontaneous retrobulbar hemorrhage is a rare presentation. The accumulation of blood in the retrobulbar space leads to increased pressure within a closed space, causing orbital compartment syndrome (OCS), and any substantial rise in pressure displaces the eye forward (proptosis). The increased pressure stretches the optic nerve, resulting in the compression of the central retinal artery, which runs inferior to the nerve in its neural sheath; impeding blood flow to the eye and resulting in vision loss. As 90 minutes of elevated intraocular pressure (IOP) can lead to permanent loss of vision [1], a retrobulbar hemorrhage is a medical emergency and requires urgent decompression by lateral canthotomy/cantholysis.

Rivaroxaban is a directly acting oral anticoagulant (DOAC) that inhibits factor Xa; it is Food and Drug Administration (FDA) approved in patients with non-valvular atrial fibrillation (AFib). AFib causes stasis of blood in the left atrium and can lead to thrombus formation, which can embolize and cause an ischemic stroke. Rivaroxaban inhibits the formation of the thrombus by inhibiting factor Xa, and the adverse risk of bleeding is low with DOAC when compared to vitamin K antagonists (warfarin). Here, we present a case of a rivaroxaban-induced spontaneous retrobulbar hemorrhage, review the pathophysiology, and discuss the diagnosis and the medical emergency of timely treatment in order to prevent permanent visual damage.

## Case presentation

A 79-year-old male with a past medical history of atrial fibrillation presented with symptoms of left eye pain, loss of vision, swelling, and redness for one week. The patient went to his ophthalmologist, who believed his current symptoms were most likely from lacrimal duct blockage, and he was advised to follow up. An appointment was scheduled with the ophthalmologist, but the patient’s visual blurriness worsened, and he eventually developed complete loss of vision, proptosis, redness, and swelling of the left eye, which made him come to the hospital. The patient denied any history of trauma to the left eye.

**Figure 1:**
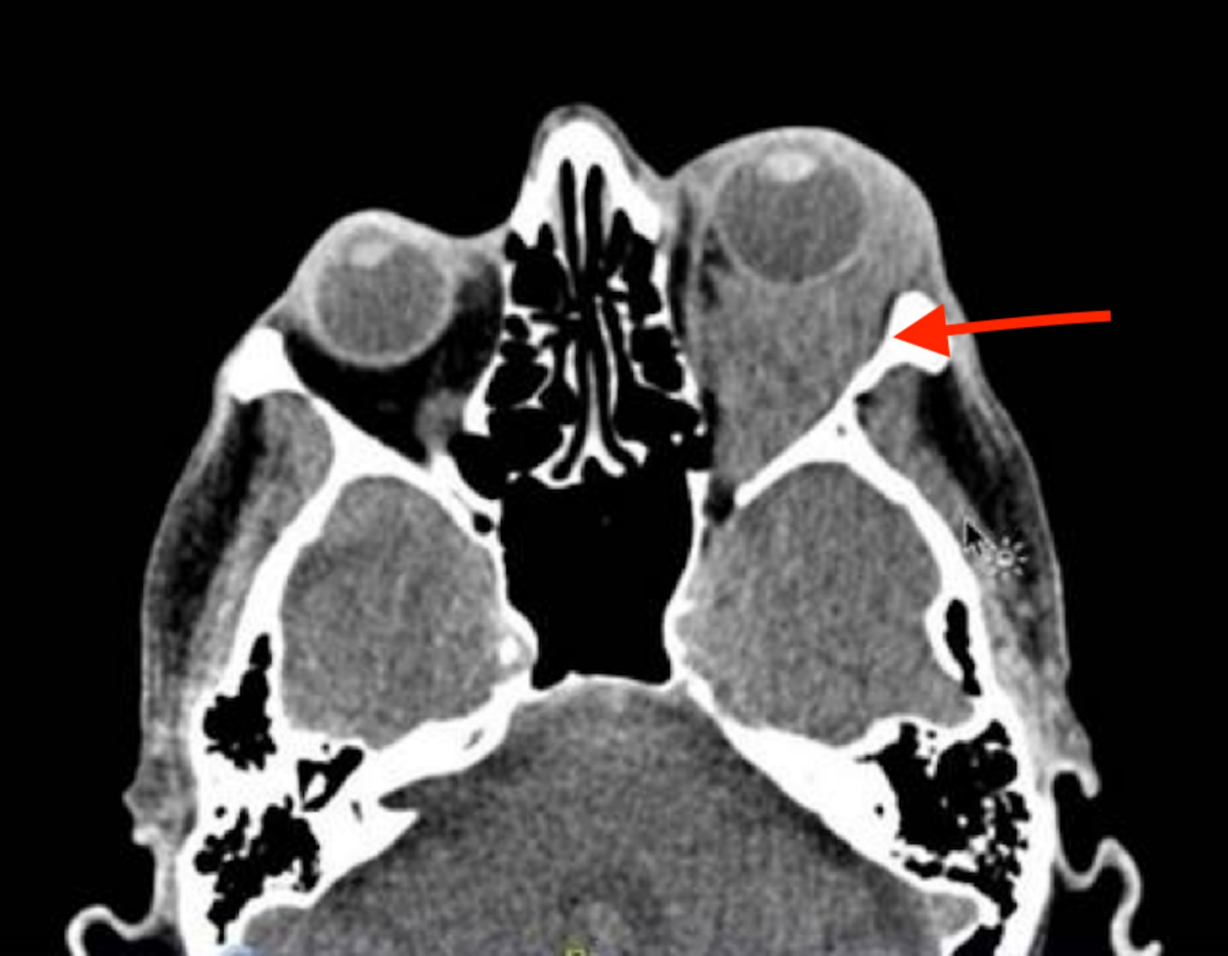
Retro-orbital hematoma and severe orbital proptosis (red arrow)

On examination, the left intraocular pressure was 47 mmHg, the pupil was mildly dilated, with no response to light, and vision was completely lost in the left eye, with redness and swelling around the left eye. Maxillofacial computed tomography (CT) scan (Figure 1) demonstrated a retroconal hematoma with severe orbital proptosis. The patient was diagnosed with a non-traumatic retro-orbital hematoma secondary to anticoagulation with rivaroxaban. Emergent ophthalmologic consultation was done and left canthotomy and cantholysis were performed. Due to the time lost initially, as the patient presented more than a week after his symptoms started developing symptoms, his vision could not be saved.

## Discussion

In a recent study, the incidence of retrobulbar hemorrhages among emergency department patients was reported to be 3%-4% with an incidence of permanent blindness of 0.14% [2-3]. The underlying etiology of non-traumatic retrobulbar hemorrhage can be broadly divided into hemorrhage due to an orbital vascular anomaly (orbital varix, lymphangioma, or arterio-venous malformation) and hemorrhage in the setting of a systemic abnormality such as bleeding disorders, uncontrolled hypertension, or septicemia [4]. The most common presenting symptoms of a retrobulbar hemorrhage include pain, diplopia (double vision), increased pressure, and loss of vision. On examination, there is usually tense or expanding proptosis, increased intraocular pressure (IOP), loss of pupillary reflexes, and optic disc or retinal pallor [5]. Diagnostic modalities include a CT scan or magnetic resonance imaging (MRI) of the orbit and direct fundoscopy to record the IOP. A value of 40 mmHg of IOP is considered the threshold above which emergent intervention is indicated [6]. Orbital compartment syndrome (OCS) is a clinical diagnosis, and treatment should never be delayed by imaging or other diagnostics [6]. Medical management consists of timolol eye drops or drugs such as mannitol, high-dose steroids, or acetazolamide [3,7]. However, the mainstay of treatment is emergent lateral canthotomy or cantholysis followed by surgical decompression in all diagnosed cases of OCS. It can be performed as a bedside procedure. An immediate complication could be an iatrogenic globe injury, hence, care must be taken in directing all instruments laterally, toward the orbital edge [6]. A spontaneous retro-orbital hemorrhage has been reported once before in a patient on rivaroxaban, where the patient's vision could be preserved due to early intervention [1]. A retro-orbital hemorrhage in a patient who underwent thrombolysis for ST-elevation myocardial infarction (STEMI) was reported by Cunneen et al. [8]. Elevated intraocular pressure for more than 60-100 minutes can cause permanent visual sequelae and complete loss of vision [1]. In a systematic review by Christie et al., it was noted that an increasing number of symptoms trended toward a prediction of blindness (p=0.092). Surgical decompression and a shorter time to treatment were each highly predictive of full recovery (p=0.024, p=0.003) and a decreased likelihood of blindness (p=0.037, p=0.045) [9].

Atrial fibrillation accounts for up to 15% of strokes in persons of all ages and 30% in patients over the age of 80 years [10]. The use of vitamin K antagonists has proved to be highly effective for stroke prevention in patients with non-valvular Afib. However, the need for frequent monitoring and dose adjustment due to increased risk of major and minor bleed makes them impractical in daily practice. Rivaroxaban was approved by the FDA in 2011 to prevent the risk of stroke and systemic embolism in patients with non-valvular atrial fibrillation. A meta-analysis study comparing the efficacy and safety of DOAC vs warfarin in patients with AFib concluded that with a decreased risk for intracranial bleeding (relative risk, 0.48, 95%; confidence interval, 0.31 to 0.74), DOACs have a favorable safety profile and significantly better treatment persistence as compared with warfarin [11].

## Conclusions

A lot of patients in this era are on rivaroxaban and other novel oral anticoagulants, and this case highlights the importance of diagnosing spontaneous orbital hemorrhage in patients on anticoagulation and intervening timely to prevent permanent vision loss.
